# Exploring medication safety in prisons: a scoping review

**DOI:** 10.1136/bmjopen-2025-103781

**Published:** 2026-03-23

**Authors:** Mohammed Alsuwat, Jennifer Shaw, Richard Neil Keers

**Affiliations:** 1 Centre for Pharmacoepidemiology and Drug Safety, Division of Pharmacy and Optometry, University of Manchester, The University of Manchester Faculty of Biology Medicine and Health, Manchester, England, UK; 2 Centre for Mental Health and Safety, Division of Psychology and Mental Health, University of Manchester, The University of Manchester Faculty of Biology Medicine and Health, Manchester, England, UK; 3 NIHR Greater Manchester Patient Safety Research Collaboration, Greater Manchester Academic Health Science Network, Manchester, England, UK; 4 Optimising Outcomes with Medicines (OptiMed) Research Unit, Pennine Care NHS Foundation Trust, Ashton-under-Lyne, England, UK

**Keywords:** Prisons, Medication Adherence, Adverse events, Prescriptions, Health Services

## Abstract

**Abstract:**

**Objective:**

Drug-related problems (DRPs), which encompass medication errors (MEs), adverse drug reactions and adverse drug events (ADEs), represent significant challenges in healthcare settings. While medication safety has been extensively studied in hospitals and primary care settings leading to development of improvement strategies, there is limited understanding of these issues within prison healthcare environments. This knowledge gap is concerning given that prisoners often have complex medication needs due to higher rates of chronic physical and mental ill health and substance use disorders compared with the general population. Time in prison presents an opportunity to provide treatment for this socially disadvantaged group, making it an important setting to optimise medication management. This scoping review aimed to understand the nature and frequency of medication safety incidents, their contributory factors and evaluate strategies for enhancing medication safety within prison healthcare environments, where unique institutional constraints and security requirements may impact safe medication use.

**Methods:**

This study conducted a systematic search across six databases to appraise the published literature from 2000 to 2023 (Embase, Medline, PsycINFO, CINAHL Plus, Cochrane and Web of Science), with reporting according to Preferred Reporting Items for Systematic Reviews and Meta-Analyses-Scoping Review guidelines. Data extraction was completed by one author, with validation of a sample by the other authors. The review included quantitative, qualitative and mixed-methods studies examining DRPs in prison healthcare.

**Results:**

The review included 42 studies on medication safety in prison healthcare, comprising epidemiological perspectives (52.4%, n=22/42), aetiological exploration of DRPs (45.2%, n=19/42) and intervention evaluation (11.9%, n=5/42). Studies were predominantly from the USA (30.9%, n=13/42) and the UK (26.1%, n=11/42). Most studies focused on infectious disease management (52.3%, n=22/42), particularly HIV treatments (42.8%, n=18/42), followed by mental health medications (23.8%, n=10/42). Non-adherence emerged as the most commonly studied DRP, reported in both epidemiological (68.1%, n=15/22) and aetiological studies (68.4%, n=13/19). Contributing factors included medication delivery problems, psychosocial factors, accessibility challenges and conflicts between healthcare delivery and security requirements. Five intervention studies were identified, with two from the USA (40%, n=2/5). These interventions included those addressing medication non-adherence (40%, n=2/5) and potentially inappropriate prescribing (20%, n=1/5), and highlighted the potential efficacy of multidisciplinary and pharmacist-led approaches in addressing medication safety challenges within prison healthcare settings.

**Conclusions:**

This represents the first comprehensive study to present a narrative report on the existing evidence regarding the epidemiology, aetiology and interventions for medication safety events in prison healthcare. While intervention studies demonstrate promising findings from multidisciplinary and pharmacist-led initiatives, further evidence is needed to address DRPs beyond medication non-adherence, guided by a deeper understanding of their contributory factors. Future studies should target preventable MEs and ADEs, as well as wider health conditions to broaden our understanding of prison-specific contributory factors to develop targeted interventions for this vulnerable population.

**STRENGTHS AND LIMITATIONS OF THIS STUDY**

- Systematic search across six major databases, with reporting according to Preferred Reporting Items for Systematic Reviews and Meta-Analyses-Scoping Review guidelines.
- Collaborative data extraction with validation by second reviewer.
- Initial citation screening by a single reviewer may have introduced selection bias.
- Exclusion of grey literature might have overlooked insights from non-peer-reviewed sources.
- Quality assessment of included studies was not feasible due to methodological heterogeneity.

## Introduction

Globally, over 11.5 million people are incarcerated, presenting significant challenges for healthcare systems responsible for their well-being.^1^ Healthcare delivery within prison environments involves complex challenges that affect not only inmates but also healthcare staff and the broader community.^2 3^ Prison overcrowding significantly compounds these challenges by straining healthcare resources, and compromising the delivery of timely medical care.^4^,^6^ Despite prisoners’ entitlement to healthcare standards equivalent to those outside prisons,^7^ implementation frequently falls short, with security protocols often delaying, disrupting or restricting access to necessary medical treatments.^8^,^10^

The prison population exhibits disproportionately high rates of chronic diseases, mental health disorders and substance use disorders compared with the general population, with studies indicating prevalence rates up to three times higher for many conditions.^11 12^ This may lead to complex health needs and result in fragmented care and inadequate health outcomes.^9 13^ Medication management within these settings presents unique obstacles, including difficulties ensuring continuity of care during transfers between facilities or on release, potential for drug diversion and complex security protocols that can impede timely medication administration.^14 15^

Correctional facilities may lag behind community healthcare settings in implementing safety initiatives,^16 17^ hampered by systemic and organisational barriers.^18^ This gap is particularly concerning given that institutional constraints and limited healthcare resources, such as insufficient medical staff, inadequate diagnostic equipment and restricted access to specialist consultations, may amplify the risks of adverse events.^19 20^

Drug-related problems (DRPs), including medication errors (MEs), adverse drug reactions (ADRs) and adverse drug events (ADEs), represent significant challenges across healthcare settings. In community settings, primary care accounts for 38.4% of MEs compared with 19.9% in secondary care settings annually in England, with an estimated cost of £98.5 million per year.^21 22^ These safety challenges may be magnified in prison settings due to multiple risk factors; complex medication regimens addressing multiple comorbidities,^23^ frequent transitions between facilities or across the community care interface,^24^ and unique adherence challenges within secure environments.^25^ While medication safety in community settings has been extensively researched,^26 27^ the literature relating to correctional facilities is disparate and may be limited, with no studies based in prisons reported in a 2019 global review of preventable harm in healthcare.^28^

This scoping review therefore addressed critical gaps in the literature on medication safety within prison healthcare systems, mapping the current evidence on their frequency, nature, contributory factors and known interventions. By adopting a scoping review methodology, this study captures a broad spectrum of evidence, identifies research gaps and sets a foundation for future research.^29 30^

## Methods

The scoping review protocol was based on the methodology framework initially established by Arksey and O’Malley, and later enhanced by Levac *et al*.^30 31^ A consultation exercise with stakeholders was not conducted due to difficulties in accessing stakeholders in the prison setting alongside evidence that stakeholder involvement has sometimes led to poorly reported outcomes in reviews.^32^

This scoping review was guided by the following research questions using the Population–Concept–Context framework:

- Population: Prisoners and incarcerated individuals.
- Concept: DRPs including their nature, frequency, contributory factors and interventions.
- Context: Prison environments.

Specific research questions:

1. What is the nature and frequency of DRPs in prison healthcare settings?
2. What are the contributory factors associated with DRPs in prison healthcare settings?
3. What interventions have been implemented and evaluated to improve medication safety in prison healthcare?

### Search and selection of studies

A database search was conducted using six electronic databases providing comprehensive coverage of the medical and allied health literature^33^: Embase, Medline, PsycINFO, CINAHL Plus, Cochrane and Web of Science.

**Figure 1:**
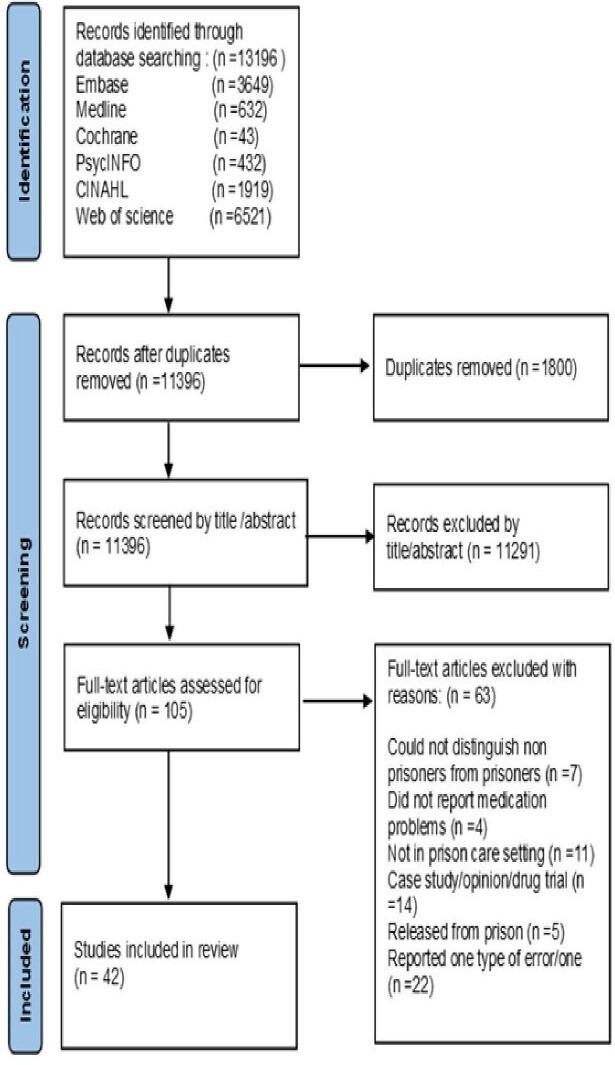
PRISMA-ScR flowchart. PRISMA-ScR, Preferred Reporting Items for Systematic Reviews and Meta-Analyses-Scoping Review.

The search terms were categorised into two themes: ‘Medication Safety Description’ and ‘Prison Setting Description’ as outlined in table 1. Several word variations related to these themes were employed. When applicable, Boolean operators (AND, OR) and truncation were used. Due to the varied syntaxes of the databases, minor modifications were made to the same search terms for each database (as shown in online supplemental file 1). No restrictions on language or topic were applied during the search to capture a broad range of literature. The search was conducted from 1 January 2000 through 15 May 2023. A Preferred Reporting Items for Systematic Reviews and Meta-Analyses-Scoping Review (PRISMA-ScR) flowchart was used to report and map the numbers at each stage of the selection process, as shown in figure 1.^29^,^31^

**Table 1:** Search terms for investigating medication safety in prison settings

Medication safety description	Prisons setting description
Drug safety	Prison*
Medication safety	Jail*
Medication error*	Secur*
Patient safety	Detain*
Adverse drug reaction*	Detention*
Adverse drug event*	Offender*
Prescribing error*	Inmate*
Medication adherence	Cellmate*
Drug compliance	Incarcerat*
Medication compliance	Criminal*
Non adheren*	Custod*
Administration error*	Defendant*
Dispensing error*	
Drug related problem*	
Drug therapy problem*	
Medication incident*	
Drug incident*	
Clinical incident*	
Incident report*	
Drug error*	
Transcription error*	
Preventable harm	
Avoidable harm	
Medication discrepanc*	
Drug discrepanc*	
Patient harm	
Drug adherence	
Medication omission*	
Drug omission*	

The definitions of medication safety terms used for this scoping review were those defined in table 2. DRPs is an umbrella term that encompasses both MEs and ADRs; including the term ensured all relevant outcome measures were captured.^34^

**Table 2:** Medication safety terms and definitions

Medication safety term	Definition
Drug-related problem	Includes unnecessary medication, ineffective medication, additional drug therapy required, dose too high/low, non-adherence and ADRs.^34^
Medication error	Any preventable event causing inappropriate medication use or patient harm while the medication is in control of healthcare professionals or the patient.^98^
Adverse drug event	An injury resulting from medication use.^99^
ADR	A harmful or unpleasant reaction resulting from the use of a medicinal product.^100^
Potentially inappropriate prescribing/medication	Prescriptions that introduce a significant risk of an adverse drug-related event when an equally or more effective alternative is available.^101^
Non-adherence	Any deviation, whether intentional or unintentional, from a prescribed medication regime.^102^

ADRs, adverse drug reactions.

On uploading all the identified citations to the software manager (EndNote 20) and eliminating the duplicates, three levels of screening were performed to assess the studies’ relevance to the eligibility criteria. First, a single reviewer (MA) evaluated the titles and abstracts independently, with any uncertainties discussed with the wider research team. The remaining papers then underwent a full-text review. At the full-text stage, explanations for the reason(s) for excluded studies were provided. During the screening and review process, all papers were scrutinised against the inclusion/exclusion criteria, which are detailed in table 3. The reasons for excluded studies at the full-text stage are explained in the PRISMA-SR flowchart (figure 1).

**Table 3:** Inclusion and exclusion criteria

Inclusion criteria	Exclusion criteria
1. Studies published from 2000 to 2023.	1. Studies based in primary or secondary care settings, including forensic wards in hospitals.
2. Studies conducted in healthcare settings within secure environments (eg, prisons or correctional facilities).	2. Case study report.
3. Studies focusing on broad medication classes (eg, antidepressants) or essential (ie, very few or no alternatives according to relevant guidelines) drug combinations used within secure settings (eg, for tuberculosis or hepatitis).	3. Studies exclusively or primarily examining specific medication side effect profiles.
4. Studies on communicable and non-communicable diseases will be included.	4. Book, dissertation, grey literature and thesis reports.
5. Reports of any medication-related problem.	5. Focused on one subtype of error (eg, wrong dose).
6. All ages and genders.	6. Studies focused exclusively on a single type of medication (eg, only amoxicillin) or a specific combination therapy where multiple alternatives exist.
	7. Abstracts with insufficient data where the full text is unavailable.
	8. Studies that were focused outside of the prison, eg, probation.

For this review, ‘broad medication classes’ were defined as therapeutic categories encompassing multiple individual medications with similar mechanisms of action or clinical applications (eg, antidepressants including selective serotonin reuptake inhibitors (SSRIs), tricyclics; antipsychotics including typical and atypical agents). Antiretroviral therapy (ART) was considered a broad medication class as HIV treatment typically involves combination therapy with multiple drug classes. Similarly, opioid agonist therapy encompasses multiple agents (eg, methadone, buprenorphine, naltrexone) used in opioid substitution treatment. Studies examining single medications only (eg, amoxicillin, metformin) were excluded as they would not provide generalisable insights about medication class-level safety challenges in prison settings.

### Charting, collating and summarising the data

The data extraction process was carried out using a standardised form provided in online supplemental file S3. MA initially extracted data from the included studies. Subsequently, members of the research team (RNK, JS) each independently reviewed a subset of seven studies to confirm the accuracy of the initial data extraction, with discussions used to resolve minor discrepancies and reach consensus.

The essential information from each study included in the review was summarised and presented in table 4, with complete individual study details provided in online supplemental table S1. These tables captured key variables such as author/year, country, study design, aim, medication classes involved, type of DRPs and any reported epidemiological, aetiological or intervention data.

**Table 4:** Summary of key characteristics of included studies (n=42)

Characteristic	Details	N (%)
Publication period	2000–2010	10 (23.8)
	2011–2023	32 (76.2)
Geographical distribution	USA	13 (30.9)
	UK	11 (26.1)
	Canada	5 (11.9)
	Ethiopia	2 (4.8)
	Spain	2 (4.8)
	Indonesia	2 (4.8)
	Iran	2 (4.8)
	Other countries*	5 (11.9)
Study design	Quantitative	24 (57.1)
	Qualitative	12 (28.6)
	Mixed methods	6 (14.2)
Research focus	Epidemiological	22 (52.4)
	Aetiological	19 (45.2)
	Interventional	5 (11.9)
DRPs	Non-adherence	27 (64.2)
	Inappropriate prescribing	8 (19.0)
	Adverse drug events	3 (7.1)
	Multiple/combined DRPs	4 (9.5)
Medication categories	Anti-infective therapy	22 (52.3)
	Mental health medications	10 (23.8)
	Other specific classes	3 (7.1)
	General/multiple classes	7 (16.7)

* Other countries: France, Norway, Taiwan, Namibia, Brazil (one study each).

DRPs, drug-related problems.

Epidemiological data were derived from quantitative studies using health records, surveys and structured assessments to examine prevalence and patterns of DRPs. Aetiological data were gathered through two approaches: quantitative analyses examining reported statistical associations between risk factors and DRPs, and qualitative methods providing rich insights into contributing factors and contexts. All findings were presented in a narrative format, emphasising key figures and findings of interest. For the analysis, data were systematically categorised and coded to assess epidemiological, aetiological and intervention-focused aspects of medication safety.

In structuring the epidemiological data, DRPs were categorised into MEs, ADEs, ADRs, potentially inappropriate prescribing/medications (PIP/PIMs) and non-adherence, as presented in table 5. The aetiological data were thematically analysed to identify contributing factors across studies, facilitating a deeper understanding of the patterns and common themes influencing DRPs in prison healthcare settings. These data are presented in table 6.

**Table 5:** Characteristics summary of (22) epidemiology studies

Study characteristic	No. of studies
Year of publication	
2000–2010	**6** (**27.3%**)
2011 onwards	**16** (**72.7%**)
Country of origin	
UK	**5** (**22.7%**)
USA	**9** (**40.9%**)
Other	**8** (**36.4%**)
DRP investigated	
Non-adherence	**15** (**68.1%**)
PIP/PIM	**4** (**18.2%**)
ADEs	**3** (**13.6%**)

ADEs, adverse drug events; DRP, drug-related problem; PIP/PIM, potentially inappropriate prescribing/medication.

**Table 6:** Characteristics summary of 19 aetiology studies

Study characteristic	Characteristic subcategory	No. of studies
Year of publication	**2000–2010**	**4** (**21%**)
	**2011 onwards**	**15** (**79%**)
Country of origin	**UK**	**8** (**42.1%**)
	**USA**	**2** (**10.5%**)
	**Other**	**9** (**47.4%**)
DRP investigated	**Non-adherence to treatment**	**13** (**68.4%**)
	**Inappropriate drug prescribing (PIP**)	**5** (**26.3%**)
	**Adverse drug events**	**1** (**5.3%**)

DRP, drug-related problem; PIP, potentially inappropriate prescribing.

Intervention data were extracted focusing on the specific aims of each study, the interventions implemented and their outcomes, and are presented in table 7. This information was analysed descriptively to report prevalence percentages and comment on the effectiveness of the interventions.

**Table 7:** Characteristics summary of five intervention studies

Study characteristic	Characteristic subcategory	No. of studies
Year of publication	**2000–2010**	**1** (**20%**)
	**2011 onwards**	**4** (**80%**)
Country of origin	**USA**	**2** (**40%**)
	**Other (Spain, Taiwan, Canada and France**)	**3** (**60%**)
Intervention delivered by	**Pharmacist lead**	**3** (**60%**)
	**Team lead**	**2** (**40%**)
Interventions	**Medication review**	**2** (**40%**)
	**Non-adherence**	**2** (**40%**)
	**Technology**	**1** (**20%**)
DRP targets	**Inappropriate drug prescribing (PIP**)	**1** (**20%**)
	**Non-adherence**	**2** (**40%**)
	**Adverse drug reactions associated with PIP**	**1** (**20%**)
	**Medication errors**	**1** (**20%**)

DRP, drug-related problem; PIP, potentially inappropriate prescribing.

## Results

### Study selection

There were 13 196 papers retrieved from the six electronic databases searched. After removing 1800 duplicates, 11 396 articles were screened by title and abstract, with 105 being taken forward to full-text review. During full-text screening, 63 papers were excluded, mainly because they reported data concerning one type of error/one specific drug (n=22), were not carried out in prison care settings (n=11) and/or were case reports, opinions or drug trials (n=14). 42 papers were included in the final review^35^,^7676^ (please see the PRISMA-ScR flowchart, presented in figure 1).

### Study distribution

Of the 42 included studies, 23.8% (n=10/42) were published between 2000 and 2010 and 76.2% (n=32/42) from 2011 onwards. Geographically, these studies predominantly originated from the USA (30.9%, n=13/42)^35^,^47^ and the UK (26.1%, n=11/42),^48^,^58^ with the remainder from Canada (n=5),^59^,^63^ Spain,^64 65^ France,^66^ Iran,^67 68^ Indonesia,^69 70^ Norway,^71^ Ethiopia,^72 73^ Brazil,^74^ Taiwan^75^ and Namibia^76^ (see table 4).

### Focus of studies

Over half of the studies (52.3%, n=22/42) focused on anti-infective therapy, specifically addressing HIV treatments (42.8%, n=18/42)^3536 39^,^42 45 47 61 62 64 68^ and tuberculosis (TB) treatments (4.7%, n=2/42),^37 75^ with one study involving both HIV and TB (2.3%, n=1/42).^67^ Hepatitis treatment was the subject of one study (2.3%, n=1/42).^63^

There were 10 studies focusing on mental health medications, including general psychotropic medications (9.5%, n=4/42),^51 54 58 65^ antipsychotics (7.1%, n=3/42),^55 57 59^ gabapentinoids (2.4%, n=1/42),^50^ benzodiazepines (BZDs) (2.4%, n=1/42),^66^ and antidepressants (2.4%, n=1/42).^38^

Specific medication classes included non-steroidal anti-inflammatory drugs (NSAIDs) (2.4%, n=1/42),^60^ opioids (2.4%, n=1/42)^71^ and nicotine replacement therapy (2.4%, n=1/42).^46^ The remaining studies (19.0%, n=7/42)^43 44 48 49 52 53 56^ investigated broader pharmacological issues, comprising general prescribing practices (five studies)^44 48 49 52 53^ and monitoring multiple medication classes (two studies).^43 56^

### Categories of drug-related problems

Non-adherence was the most commonly investigated DRP (64.2%, n=27/42),^3536 38^,^40 42 43 45^ followed by PIP/PIM (19.0%, n=8/42)^48 49 51 53 54 58 60 72^ and ADEs (7.1%, n=3/42).^37 52 75^ MEs were studied in one study (2.4%, n=1/42).^44^ Few studies investigated multiple DRPs: non-adherence combined with ADRs (2.4%, n=1/42),^41^ PIP combined with ADRs (2.4%, n=1/42)^66^ and PIP combined with drug–drug interactions (2.4%, n=1/42).^50^

### Methodological approaches

Quantitative methods were used in 57.1% (n=24/42) of the included studies. Among these, health records were the most commonly used source of data, featuring in 21 studies.^36^,^3840 41 43^ Surveys were employed in one study (2.3%, n=1/42),^46^ and structured questionnaires were used in two studies (4.8%, n=2/42).^64 73^

Qualitative methodologies were used in 28.6% (n=12/42) of the studies. Within this category, interviews were the predominant method, conducted in eight studies (19.0%, n=8/42).^42 48 53 58 62 71 72 76^ Focus groups were used in two studies (4.8%, n=2/42),^67 68^ and another two studies (4.8%, n=2/42)^39 57^ employed surveys primarily with free-text responses.

Mixed methods approaches accounted for 14.2% (n=6/42) of the studies. Three studies (7.1%, n=3/42) combined health record reviews with interviews,^35 49 55^ two studies (4.8%, n=2/42) used interviews and questionnaire data,^56 70^ while one study (2.3%, n=1/42) employed both quantitative and qualitative analysis of incident reports.^52^

### Data insights

Epidemiological data were reported in 52.4% (n=22/42) of the studies,^35^,^3840 41 43 45 47 49^ offering important insights into the prevalence and characteristics of DRPs. Aetiological factors were explored in 45.2% (n=19/42) of the studies.^3942 48 49 52 53 55^,^58 62 64 67 68 70^ Four studies (9.5%, n=4/42) combined both epidemiological and aetiological data.^49 52 64 73^ A smaller subset of studies (11.9%, n=5/42) focused on evaluating medicines management interventions.^44 46 60 65 66^

This flowchart illustrates the systematic search and selection process, reported according to PRISMA-ScR guidelines for the scoping review on medication safety in prisons. Records were identified through database searching (n=13 196) across six electronic databases: Embase (n=3649), Medline (n=632), Cochrane (n=43), PsycINFO (n=432), CINAHL Plus (n=1919) and Web of Science (n=6521). After removing duplicates (n=1800), 11 396 records were screened by title and abstract, with 105 proceeding to full-text review. 63 papers were excluded during full-text screening, resulting in 42 studies included in the final review.

### Epidemiology of drug-related problems

Epidemiological studies comprised 52.4% (n=22) of the initial sample of 42 studies. These studies were predominantly published post-2010 (72.7%, n=16/22) and originated mainly from the USA (40.9%) and the UK (22.7%). Quantitative approaches dominated, with health or medical record review being the primary data collection method in fourteen studies (63.6%, n=14/22). Non-adherence was explored in 68.1% of studies (n=15/22), while potentially inappropriate drug prescribing (PIM/PIP) was reported in 18.2% of the studies (n=4/22) and the remaining studies examined ADEs (13.6% (n=3/22)).

### Non-adherence

Non-adherence prevalence rates varied significantly across drug classes and conditions. Multiple studies have examined this issue across different prison populations and medication types. In one study in a USA prison, a third of the 2554 inmates with depressive disorders were non-adherent to antidepressant therapy. The median compliance score (where 1.0 represents perfect compliance) was 0.79. Adherence was higher among those prescribed tricyclic antidepressants (0.80) compared with SSRIs (0.75), with males adhering marginally better than females (0.80 vs 0.74) and compliance increasing with age, peaking at 0.86 for inmates aged 50 and older.^38^

A study in British Columbia included 12 102 offenders diagnosed with schizophrenia, which represented 31% of the prison population studied. They examined medication adherence using the medication possession ratio (MPR), which is a measure of the proportion of days an individual has medication available. The overall mean MPR was 0.41, indicating that inmates had their medication available for 41% of prescribed days. Only 35% of inmates met the recommended threshold of having medication available for at least 80% of prescribed days during the first 120 days of follow-up. Adherence declined further over time, with only 27% meeting this threshold in the subsequent 120-day period.^59^

To better understand medication adherence patterns in context, researchers have conducted comparative analyses between correctional and community settings for people living with HIV. The Seek, Test, Treat and Retain cohort study comparing criminal justice-involved persons (n=414) to routine care patients (n=11 698) found that incarcerated individuals had significantly different adherence patterns. Incarcerated individuals were more likely to have a detectable viral load (VL) (26% vs 12%, p<0.001), but had lower rates of optimal adherence scores≥95% (59% vs 70%). Each 10% increment in adherence was associated with a 27% reduction in VL among criminal justice-involved persons (relative VL=0.73, 95% CI 0.65 to 0.83, p<0.01) and a 34% reduction among routine care patients. Similar associations were found using binary measures of viral suppression, where optimal adherence (≥95%) was associated with significantly lower odds of having a detectable VL in both populations.^47^

A study in Ethiopian prisons demonstrated varying adherence rates depending on the measurement method used. When comparing prisoners with HIV to community-based HIV patients, pharmacy refill records showed higher adherence among incarcerated individuals (89% pharmacy refill adherence) compared with those in the community (75%). However, when using direct dose monitoring, the difference was smaller, with prisoners showing 81% adherence compared with 83% in community patients.^73^ A study in São Paulo prison units (n=67) demonstrated relatively high short-term adherence rates, with 80.6% of participants reporting no missed doses of ART in the previous 7 days. The study found that 91% of inmates adhered to prescribed tablet quantities, though 76.1% reported taking medications at irregular hours.^74^

There were observed discrepancies between different adherence measurement approaches, as demonstrated in both the Ethiopian study^73^ and a USA prison study.^36^ In the USA study, while self-reported adherence reached 100%, objective measures showed lower rates; 90% by pill count and 86% by electronic monitoring caps. This finding indicated that 68% (21/31) of participants fell below the required adherence threshold of ≥90% for optimal treatment outcomes, despite being in a controlled prison environment where medication access was supervised.^36^

Significant geographical variations were noted in adherence outcomes across studies. An Indonesian prison study reported that while 73.3% of participants used ART, only 25.5% achieved optimal adherence based on self-rating scales and clinical measures. This contrasts with USA prison facilities, where studies reported adherence rates exceeding 85% using self-report questionnaires and pharmacy refill data.^40 69^ These disparities highlight the importance of considering regional resources and healthcare systems when interpreting adherence patterns across different prison populations, even when similar measurement tools are employed.

### Potentially inappropriate prescribing

A national study of prescribing in English prisons found that undocumented or unapproved indications for prescriptions, not listed in the British National Formulary, were recorded in a third (34.7%, 95% CI 32.5% to 37.0%) of cases, most commonly for low mood and personality disorder.^54^

Among 1941 sentenced men arriving at a UK prison between February 2017 and November 2018, 634 (33%) were prescribed psychoactive medications. Of these, 474 (75%) required prescription changes due to appropriateness concerns (PIP) and/or safety issues. At reception, despite prior medicines reconciliation, 295 (46.5%) received changes, with 56 (8.8%) changes due to appropriateness concerns alone and 194 (30.6%) due to both appropriateness and safety concerns. During follow-up, an additional 275 (43%) required changes, with 69 (10.9%) due to appropriateness concerns alone, 163 (25.7%) due to safety concerns alone and 43 (6.8%) due to both appropriateness and safety issues.^51^

Further evidence of PIP was found in a UK prison study examining gabapentinoid prescribing. Among the study population, 34% of prescriptions lacked documented indications for the drug. Of those with documented indications, 50% were for unlicensed uses. The study also revealed inadequate documentation of drug safety concerns; while 47% of patients were coprescribed opioid substitution therapy (methadone or buprenorphine) with gabapentinoids, only 22% of these cases had the potential harmful interaction documented in patient records.^50^

Among older prisoners, 25.8% were prescribed medications with medium or high anticholinergic activity, while 46.3% were prescribed BZDs, Z-drugs or sedating antihistamines for longer than 1 month.^49^ Additionally, 39.1% of patients prescribed antipsychotics for over 12 months lacked metabolic monitoring during the previous year.^49^ This data comes from a 2021 cross-sectional study that analysed electronic medical records from two UK male prisons, using search protocols within the prison electronic health record system to examine prescribing safety indicators.

### Adverse drug events

A study of latent TB infection treatment in the USA found that among 1211 incarcerated patients receiving rifampicin and pyrazinamide, 13.4% experienced ADEs, with hepatotoxicity being the predominant adverse event. Several risk factors were statistically associated with increased hepatotoxicity: older age (adjusted OR (AOR)=0.97, p=0.01), abnormal baseline aspartate aminotransferase levels (AOR=0.72, p=0.02) and excess alcohol use (OR=2.1, p=0.03).^37^ A similar pattern was observed in a study from Taiwan of 373 prison inmates, where 12% experienced adverse events leading to treatment discontinuation with 6-month isoniazid (6H) treatment, with hepatotoxicity (8%) being the most common.^75^ Both studies noted that hepatitis C virus (HCV) infection was an independent risk factor for ADEs (p=0.02) among a population with high hepatitis prevalence (hepatitis B virus 13%, HCV 21%).^37 75^

### Aetiology

Aetiological studies comprised 45.2% (n=19/42) of the included studies. These studies were predominantly published post-2010 (79%, n=15/19), and originated mainly from the UK (42.1%, n=8/19) and the USA (10.5%, n=2/19). 12 studies used qualitative methods, with interviews being the primary method in 8 studies, focus groups in 2 studies and surveys with free-text responses in 2. Mixed methods approaches were used in four studies, while three studies used quantitative approaches to examine statistical associations between risk factors and DRPs. Non-adherence to treatment emerged as the most frequently investigated DRP, with 13 out of 19 studies (68.4%) focusing on this issue. PIP was the second most common DRP investigated, being addressed in 5 out of 19 studies (26.3%). The remaining study examined ADEs (5.3%).

### Factors leading to non-adherence

Depressive symptoms in inmates were associated with a 74% lower likelihood of dose adherence compared with those without depressive symptoms in one study from Ethiopia published in 2020, which used structured questionnaires, self-reported adherence and pharmacy refill records.^73^ HIV/AIDS-related stigma and discrimination were observed in an Iranian study published in 2014, involving focus group discussions where inmates reported feeling ashamed or fearful of their diagnosis, causing them to avoid taking medication openly.^67^ The fear of being judged or treated poorly by both inmates and prison staff was reported by one study from the USA (2009), leading some women to hide their HIV status, impacting adherence negatively.^42^ Social isolation due to HIV/AIDS-related stigma was also described as leading to a lack of support from peers or family, which in turn decreased adherence.^42 67^ Substance abuse, in particular, was reported to interfere with adherence to a daily medication regimen due to cognitive impairment and chaotic lifestyles.^42^

The structured environment of a jail was reported to make adherence to a medication regimen challenging, particularly when it involved attending scheduled directly observed therapy (DOT) sessions. In one USA study,^39^ where surveys were administered to 102 inmates, prisoners revealed that DOT often led to the inadvertent disclosure of their HIV status, resulting in stigma and discrimination from other inmates and staff, with subsequent non-adherence.^39^ In this San Francisco survey study examining medication administration preferences, inmates reported that self-administered therapy would be more convenient, allowing them to incorporate medication-taking into their daily routines without disruption and stigma.^39^ Convenience and the ability to establish a routine were significant factors, with 67.6% (n=69/102) of inmates believing that self-administration would help them establish a medication-taking routine essential for maintaining adherence both during incarceration and after release. Additionally, the study found that medication theft and misuse were factors impacting adherence, suggesting that appropriate safeguards were necessary to mitigate these risks.^39^ In a separate 2009 USA study using in-depth interviews with 12 female inmates in state prison, some women expressed a preference for the existing Keep Own Prescription systems for greater control and privacy, allowing them to retain their medications and self-administer them without direct supervision by healthcare staff. This approach, similar to a form of ‘in possession’ medication management seen in the UK, was favoured by those who valued independence in managing their medication schedules. Conversely, DOT was deemed beneficial for individuals who required a more structured environment to maintain adherence.^42^

Other aspects of the prison environment, including unplanned transfers of patients to other prisons, units or wards, and transfers to court or police stations, were reported to disrupt the continuity of care and access to medications. Frequent transfers during the pretrial and sentencing phases further exacerbated this issue.^62 67^ A qualitative study from Canada published in 2009 reported that short-term interruptions in medication access, lasting approximately 1–3 days, were common during these transfers due to systemic delays in verifying prescriptions and procuring medications. Additionally, participants highlighted that local police facilities, where detainees were typically held for 1–3 days, lacked provisions for dispensing HIV medications, resulting in significant barriers to adherence. These delays were reported to often extend beyond initial detention periods due to poor communication between correctional facilities and healthcare providers, leaving some individuals without access to essential HIV medications throughout their incarceration or until they were released. Such interruptions were reported to negatively impact adherence and treatment continuity, as participants sometimes chose to reinitiate therapy only after returning to community care.^62^

Challenges within institutional healthcare systems such as medicine availability, lockdowns and administrative delays were also reported across three studies to negatively impact adherence.^39 49 62^ Delays in verifying prescriptions, procuring medications and the actual delivery to inmates were common issues. In one study using interviews with 12 HIV-positive male injection drug users in Canada,^62^ reliance on institutional healthcare staff to order and dispense HIV medications introduced additional challenges, with many participants reporting experiencing medication interruptions due to organisational delays. The perceived quality of HIV care in prisons was often seen as inferior to community care, primarily due to the limited HIV-specific knowledge of institutional healthcare staff which affected their ability to manage and adjust treatment regimens appropriately.^62^ Missing clinic appointments significantly increased the risk of non-adherence as reported in a 2022 Ethiopian study using structured questionnaires, self-reported adherence and pharmacy refill records.^73^ In contrast, positive interactions with healthcare providers were seen to facilitate adherence in a 2008 UK study involving inmate surveys with free-text responses, while negative experiences hindered it.^57^

Harsh conditions within the prison, including violence, psychological humiliation and general despair, contributed to a lack of motivation among prisoners to maintain their health and adhere to ART.^49^ Personal characteristics such as age and ethnicity, prior medication use, insight into treatment needs, the structure of the prison environment and perceptions of medication side effects influenced adherence.^57 58^ Managing medication side effects effectively was crucial, as both the presence and perception of side effects significantly negatively influenced adherence.^57^ Some prisoners tolerated side effects, such as drowsiness, lack of appetite and extrapyramidal symptoms, to gain the benefits of symptom management, while others discontinued medication due to such distressing side effects.^55 57^

### Factors leading to potentially inappropriate prescribing

Multiple factors were identified as contributing to PIP practices in prison settings. A qualitative study conducted in the UK highlighted the lack of clinical governance structures, inconsistent guidelines and absence of standardised prescribing strategies as significant contributors to PIP.^48^ Interviews with prison healthcare staff revealed challenges in following clear protocols due to limited oversight, resulting in variability in prescribing practices. These issues significantly impacted medication safety and the effectiveness of treatment.^48^

Prison-specific structural barriers, such as capacity and security categorisations, high turnover rates of the prison population and restricted operational processes, further complicated appropriate prescribing. Limited consultation time and rigid scheduling, driven by security constraints, were frequently reported as contributing to prescribing decisions made under suboptimal circumstances. These barriers increased the risk of prescribing errors or inappropriate medication selection.^48 72^ Staff shortages, insufficient training and delays in obtaining security clearance for healthcare providers were significant challenges contributing to PIP, as revealed through qualitative in-depth interviews with HIV-infected prisoners and stakeholders in Ethiopia in a 2021 study.^72^ In a UK study, prison healthcare staff frequently reported feeling underqualified to address the diverse and complex needs of incarcerated individuals, including managing chronic illnesses, polypharmacy and mental health conditions.^48^ This inadequacy, compounded by a lack of access to specialised support, posed additional risks for inappropriate prescribing.^48 53^

Healthcare providers in prisons reported facing information technology (IT)-related limitations, such as a lack of integrated records systems, as identified in a 2022 UK study using interviews with healthcare staff and pharmacists.^48^ Similarly, in Ethiopia, poor links between prison healthcare services and external health facilities meant that healthcare staff had incomplete patient histories.^73^ These limitations increased the risk of prescribing errors, particularly in complex cases involving multiple conditions or medications, as healthcare providers lacked comprehensive information needed for safe prescribing decisions.^48 73^

A study conducted in the East of England prisons between 2010 and 2011 explored the perspectives of healthcare staff and prisoners on the purpose of psychotropic medication use in prisons. The qualitative study involved interviews with 17 patients and 16 staff members and found that psychotropic medications were often reported to be discontinued on prisoner reception, with 47% of prescriptions stopped without documented justification. This was said to lead to frustration among prisoners and raised concerns about the continuity of care. Moreover, the study highlighted the high prevalence of mental health issues in prisons, noting that one in seven prisoners had a psychotic disorder or major depression. Staff expressed concerns over the reliance on psychotropic medication while prisoners pointed to insufficient mental health support, which led to increased dependence on medications to cope with prison life.^58^

Similarly, the findings from a 2018 qualitative study conducted in the UK using semistructured interviews with healthcare staff to explore prescribing practices highlighted systemic challenges, including resource constraints and insufficient training, which compounded difficulties in implementing appropriate prescribing practices.^53^ Additionally, prescribing guidelines aimed at preventing medication diversion and misuse emphasised prescriber responsibility and embedded assumptions about the untrustworthiness of imprisoned individuals. These systemic framings fostered a climate of distrust, leading to restrictive prescribing practices that could exacerbate DRPs and weaken therapeutic relationships between healthcare providers and patients.^53 58^

A 2021 qualitative study examining prescribing barriers for ART among HIV-infected prisoners in South Ethiopia employed in-depth interviews with seven HIV-infected prisoners (five male and two female) and 11 stakeholders including health staff, prison officers and HIV programme coordinators. The study identified multiple systemic barriers to appropriate prescribing and treatment initiation, including insufficient testing facilities, limited availability of HIV test kits and delayed diagnosis and treatment. Health staff faced challenges in timely care linkage due to delayed test result communication and inadequate training in pretest and post-test counselling. The uncooperative prison security system often denied or delayed inmates’ visits to external health facilities and breached privacy, discouraging ART initiation due to stigma. Additionally, public disclosure during healthcare procedures and negative attitudes from prison officers further deterred treatment initiation.^72^

### Interventions

Interventional studies comprised only 11.9% (n=5/42) of the initial sample of 42 studies. These studies, predominantly published post-2010 (80%, n=4/5), originated from the USA (40%, n=2/5)^44 46^ but included studies from Spain, Canada and France (60%, n=3/5).^60 65 66^ These interventions primarily involved multidisciplinary teams of healthcare professionals—physicians, nurses and pharmacists—with three^44 60 66^ specifically led by pharmacists. They addressed a range of DRPs, including non-adherence (n=2/5, 40%),^46 65^ PIP (n=1/5, 20%),^60^ PIP associated with ADRs (n=1/5, 20%)^66^ and MEs (n=1/5, 20%).^44^

### Non-adherence

In Spain, the Treatment Adherence Programme (TAP), which combined psychoeducation and motivational interviewing sessions, aimed to improve adherence to psychopharmacological treatment among prisoners with mental health problems. Participants were randomly assigned to either the TAP group or the treatment as usual (TAU) group. The TAP group showed significant increases in mean adherence scores, measured using the validated Morisky Medication Adherence Scale-8, 3 months postintervention (3.7, 95% CI 3.5 to 3.9) and 9 months postintervention (3.9, 95% CI 3.6 to 4.2), with significant differences from the TAU group (p=0.002 and p=0.004, respectively).^65^

A smoking cessation programme for female prisoners in the USA tested adherence to a combined intervention of nicotine replacement therapy (NRT) and behavioural counselling. Over 10 weeks, adherence rates, assessed through weekly self-reports and validated by staff observation records, were 66.8% for NRT alone, 51.2% for counselling alone and only 45.0% for both components combined. Adherence to NRT was notably higher among participants with previous quit attempts (p=0.04) and high baseline cigarette consumption (p=0.03). Participants who adhered to both components were significantly more likely to sustain self-restraint post-treatment.^46^

### Potentially inappropriate prescribing

In France, a pharmacotherapy programme was implemented to address ADRs associated with PIP of BZDs among 226 prisoners at a correctional facility. The programme began in the year 2000, with a baseline mean daily BZD dose of 42 mg diazepam equivalent (DE) per day. By 2004, the mean daily BZD dose had decreased to 30 mg DE per day, a 29% reduction (p<0.001), as measured through systematic review of pharmacy dispensing records and computerised prescriber order entry data. This reduction was achieved through monthly meetings and systematic medication reviews by pharmacists and psychiatrists. In 2008, the mean daily dose was 31 mg DE per day, a 26% reduction from the baseline (p<0.001), showing sustained adherence to the prescription guidelines. By 2012, the dose increased slightly to 35 mg DE per day, a 17% reduction from baseline, though not statistically significant (p>0.05). This increase followed guideline updates in 2009 that raised the maximum daily dose. By 2016, the mean daily dose had decreased to 29 mg DE per day, a 31% reduction from baseline (p<0.001). Subgroup analyses revealed significant dose reductions among patients on opioid maintenance therapy and those initially prescribed high doses.^66^

In Canada, clinical pharmacists aimed to reduce PIP of oral NSAIDs among prisoners. At the start, 70% of the 53 included patients were on oral NSAIDs. After a 3-month intervention, this number dropped to 47%, a 32.4% reduction (95% CI 17.3% to 47.5%). The intervention also optimised NSAID formulations, shifting from diclofenac 10% gel to diclofenac 2.32% gel. Pharmacists addressed 153 DRPs across 51 patients, averaging three interventions per patient, including medication adjustments, initiations, discontinuations, adherence education and non-drug interventions. All pharmacist recommendations were accepted by primary care physicians.^60^

### Medication errors

In one study from the USA, implementation of an automated check-and-sortation system in a prison aimed to reduce MEs and improve clinical interventions by pharmacists. Clinical interventions, documented through the pharmacy department’s quality assurance programme records, increased from 396 to 1075 per 100 000 medication orders, a 171% rise within the first year of implementation (1999–2000). Dispensing errors decreased from 6.3 to 4.1 per 100 000 orders in the first 6 months, a 35% reduction and further decreased to 3.3 per 100 000 orders by the end of the first year, a total decrease of 48%. Identified filling errors, tracked through the automated system’s error detection logs, increased from 224 to 256 per 100 000 orders, a 14% rise, indicating enhanced error detection. The system allowed pharmacists to focus more on clinical activities, with therapeutic interventions increasing from 16.5% to 20.9% and compliance interventions from 2.1% to 11.7%.^44^

## Discussion

This scoping review used a systematic approach to explore medication safety within prison healthcare, analysing a total of 42 studies published over a 20-year time period. There has been increased research attention in this area since 2011 (76.2%), reflecting growing recognition of medication safety issues.^17^ Our findings revealed a distinct geographical concentration of research, with the majority of studies originating from the USA (30.9%, n=13/42)^35^,^47^ and the UK (26.1%, n=11/42).^48^,^58^ There was limited research representation from other regions, particularly low- and middle-income countries, where prison healthcare faces additional constraints such as resource limitations, overcrowding and inadequate healthcare infrastructure.^10 12^ This limited geographical representation restricts our ability to develop globally applicable solutions for medication safety in correctional settings.^77^

Our review revealed a significant imbalance in medication safety research focus; 64.2% of studies examined medication adherence, most explored the epidemiology of medication safety events (n=22/42, 52.4%) and the majority considered infectious disease management (52.3%), and in particular HIV treatments (42.8%). This resulted in observed research gaps in other areas; for example, only 11.9% of included studies examined interventions to improve medication safety,^44 46 60 65 66^ and few explored preventable medication safety events with 2.3%^44^ of studies including MEs and 7.1%^37 52 75^ ADEs.

When examining epidemiological studies (n=22), the methodological landscape was dominated by health and medical record reviews (63.6%, n=14/22), where the focus was disproportionately on infectious disease and mental health management. This focus may in part reflect the disproportionate prevalence of infectious diseases and mental health problems in correctional settings.^11 12^ Incarcerated populations may often originate from socially disadvantaged backgrounds with complex health needs and limited contact with community health services,^13^ making prisons important settings for addressing both individual care and broader public health challenges.^2 3^

Non-adherence emerged as the most commonly studied medication safety concern in our review. Studies included in this review revealed evidence of lower rates of adherence in incarcerated individuals compared with those outside prison.^36 47 73^ Given the supervised nature of these environments, where theoretically medication administration could be more controlled than in community settings, this potentially highlights a missed opportunity for improved therapeutic outcomes. Multiple prison-specific factors contributed to non-adherence, including institutional constraints such as staff shortages,^58^ rigid medication administration schedules,^42^ privacy concerns during medication distribution,^39 67 68^ stigma associated with certain conditions,^42 67^ frequent transfers disrupting medication continuity^62 67^ and psychosocial factors including depression.^73^ The influence of comorbid mental illness on adherence may highlight intervention opportunities when viewed at scale, given the elevated prevalence of mental health conditions in prison populations.^10 42^ Despite identifying these unique challenges, our review found only two intervention studies (40% of all interventions) addressing non-adherence,^46 65^ and while they were reported to be successful, these interventions did not comprehensively address the full range of barriers identified here, suggesting a disconnect between known causes and implemented solutions.

An important methodological consideration when interpreting adherence data from prison settings relates to how institutional medication management systems may affect adherence measurement validity. Multiple studies in our review assessed medication adherence using various community-based measures including MPR,^59^ self-reported adherence questionnaires and scales,^36 40 47^ pill counts,^36^ electronic monitoring caps^36^ and pharmacy refill records.^40 69 73^ However, some adherence measures may be associated with limitations when applied in prison environments where DOT,^35 36 39 40^ in-possession policies^56^ or restricted medication handling can be common practice.

Reported adherence rates may therefore be reflective of institutional policies than actual patient adherence. For example, in correctional facilities, as in hospitals, patients do not refill prescriptions and, with the exception of medicines kept ‘in possession’, healthcare staff control medication administration according to security protocols. Some medications may also be kept as ‘stock’ in clinic rooms rather than as named dispensing to individual patients, which may make tracking adherence via individual patient dispensing records more challenging.^35 36^ Known issues with medication trading and abuse in prison settings are another complicating factor, with concerns about diversion potentially affecting both prescribing decisions and adherence measurement approaches.^39 53^

Research in correctional settings has demonstrated that even when DOT is implemented, different adherence measurement methods yield different results for the same patients, with medication administration records showing higher adherence than electronic monitoring caps for identical medication regimens.^36 40^ Future research in correctional settings should consider the impact of institutional medication policies when developing and selecting adherence measures, potentially favouring methods that account for these and which reflect actual medication usage patterns by patients.

Despite their elevated prevalence in prison healthcare, the narrow focus on infectious diseases and mental health illness risks overlooking medication safety challenges affecting the broader range of chronic health needs in prison populations. Recent systematic reviews have highlighted that prisoners have significantly higher rates of non-communicable diseases, including cardiovascular disease, diabetes and respiratory conditions, which may become even more prevalent as the prison population ages. Recent studies indicated prevalence rates of cardiovascular disease of 38%, chronic obstructive pulmonary disease of between 4% and 18%, and diabetes of 14% among incarcerated individuals.^11^ ^78^ Evidence from primary care and hospital settings suggests these conditions carry substantial medication safety risks. With drug-related incidents accounting for approximately 25% of preventable patient harm,^28^ with MEs being particularly common in healthcare settings generally.^26^ Studies have found that insulin administration errors can lead to severe harm in up to 33% of cases.^79^ Respiratory medications present different challenges, with incorrect inhaler technique affecting 55%–80% of patients according to systematic review data, potentially leading to therapeutic failure.^80 81^ Despite this disease burden, our review found minimal research exploring medication safety for these conditions in prison settings, where unique contextual factors such as supervised medication administration systems, security protocols and resource limitations could further compromise appropriate medication use. This represents a substantial evidence gap for clinicians and policymakers.

PIP was also found to be a frequent occurrence in prison healthcare, with commonly studied medication groups including psychotropic agents and gabapentinoids. These medications are significant not only in terms of their prevalence but also their association with preventable harm, and medication diversion in prison populations.^82 83^ Psychotropic prescribing in UK prisons has been documented at rates six times higher than in the general population,^54^ while gabapentinoids, which pose substantial risks for misuse and dependence.^50^ The causes of PIP identified in our review included inadequate clinical governance structures,^48^ insufficient prescriber training in prison-specific healthcare,^48 72^ limited consultation time due to security constraints^48 53^ and prescribing driven by security concerns rather than clinical need.^53 58^ Similarly, the observation that 75% of psychoactive medications required changes due to appropriateness or safety concerns on reception to prison^51^ further illustrates the magnitude of this problem. This rate was substantially higher than the 8.5%–10.2% of prescriptions requiring intervention in acute hospital settings.^84^ High rates of undocumented indications (34.7%)^54^ and documented unlicensed uses (50%)^50^ are concerning given that standard prescribing guidelines mandate clear documentation of clinical justification and systematic monitoring.^85^ Notably, only one intervention study^60^ specifically addressed PIP, suggesting a significant gap between identified problems and tested solutions. Our findings suggest opportunities for prison healthcare to implement interventions designed to improve appropriate prescribing. The Pharmacist-led INformation technology intervention for medication ERrors and Salford Medication safety dASHboard studies demonstrated that pharmacist-led information technology interventions were more effective than simple feedback at reducing MEs in general practices (OR 0.51, 95% CI 0.34 to 0.78).^86 87^ Recently, a multidisciplinary intervention using prescribing safety indicators specifically tailored for UK prisons was evaluated, demonstrating feasibility and acceptance among prison healthcare staff. This approach leveraged a suite of prescribing safety indicators to identify PIMs, including high-risk psychotropics, and employed multidisciplinary teams to review and optimise prescribing practices in prison settings.^88^

This review has identified a limited volume of epidemiological and aetiological research focused on preventable MEs^44^ and ADEs,^37 52 75^ despite their significant impact on patient safety and position as a global healthcare priority. Preventable medication-related harm has been identified as a central focus of major patient safety initiatives worldwide, including the landmark ‘To Err is Human’ report in the USA,^89^ key UK healthcare quality initiatives informing the National Health Service Patient Safety Strategy^21^ and WHO’s Global Patient Safety Challenge ‘Medication Without Harm’.^22^ This contrasts sharply with community healthcare settings, where robust incident reporting systems, systematic safety reviews and data collection frameworks have been established to detect, analyse and prevent medication safety incidents. In the UK alone, primary care accounts for 38.4% of MEs compared with 19.9% in secondary care settings annually, with an estimated cost of £98.5 million per year.^21 22^ While prisons theoretically have access to the same reporting frameworks as community settings, our findings suggest these may be underused or face implementation barriers specific to correctional environments. A systematic review and meta-analysis of 70 studies involving 337 025 patients published in 2019 found that approximately 1 in 20 patients experienced avoidable healthcare-associated harm in general healthcare settings, but comparable data for prison populations was notably absent.^90^ Similarly, a 2020 systematic review and meta-analysis of 81 studies involving 285 687 patients found that the pooled prevalence for preventable medication harm was 3% (95% CI 2% to 4%) across healthcare settings, yet none of these studies examined prison healthcare.^91^ This represents a significant knowledge gap, particularly concerning given the higher prevalence of complex health conditions and medication regimens among incarcerated individuals which may precipitate error.^11 12^

Our aetiological analysis benefited significantly from the inclusion of qualitative methodologies (28.6% of studies), which provided richer and more contextually nuanced insights than purely quantitative approaches examining statistical associations between risk factors and medication safety outcomes. Where quantitative studies identified correlations, qualitative investigations illuminated the underlying mechanisms and lived experiences creating these relationships. For example, quantitative data showed higher non-adherence rates among patients with depressive symptoms^73^ (74% lower likelihood of dose adherence), while qualitative interviews revealed how this relationship was mediated through reduced motivation and self-efficacy within the restrictive prison environment.^67 72^ Mixed-methods approaches combining quantitative measurement with qualitative exploration have been widely advocated in patient safety research,^49 52 89^ with systematic reviews recommending integrated methodologies that capture both the incidence and causal mechanisms of medication safety events.^28^ While these aetiological findings provide valuable insights, we need further understanding of how particularly preventable medication safety events manifest across different prison systems and healthcare models using theory-driven approaches.

While evidence of intervention effectiveness in prison healthcare is limited, systematic reviews of ME reduction strategies in community settings have demonstrated that targeted interventions can significantly reduce MEs rates by 34%–66% in outpatient and ambulatory settings.^92^ Our review showed promising approaches that could be built on for future initiatives. The TAP combining psychoeducation and motivational interviewing successfully improved adherence to psychotropic medications among prisoners with mental health problems,^65^ leveraging the unique environment of prisons where diverse healthcare and security staff may collaborate to support patient care. This multidisciplinary approach has also been seen in a recently published qualitative evaluation of a prescribing safety indicator-based intervention in prison healthcare settings.^88^ Pharmacist-led interventions also showed positive outcomes, including reductions in BZD doses through systematic medication reviews^66^ and reducing inappropriate NSAID prescribing.^60^ These findings align with broader evidence from community settings, where clinical pharmacy services have been shown to reduce medication errors by up to 30% and inappropriate prescribing by 35%.^93 94^ Our review found limited evidence of technology-enabled interventions in prison settings, with one study showing a 35% reduction in dispensing errors through automated systems.^44^ While Electronic Medication Management Systems (EMMS) have been successfully introduced in tertiary hospitals through patient-centric approaches,^95^ their application within correctional facilities presents distinct challenges.^49^ Compared with acute hospitals, implementation of EMMS in correctional environments requires additional consideration of unique infrastructural limitations such as restricted internet access, legacy IT systems, incompatible electronic health records between prison and community healthcare, limited physical space for technology installation and security concerns about potential misuse of electronic equipment.^48 49 53^ These limitations, combined with rigorous security protocols, create substantial barriers to implementing digital health solutions that have proven effective in other settings.^96^

### Strengths and limitations

The main strengths of this scoping review are primarily its systematic approach to aggregating a broad spectrum of evidence on DRPs in prison healthcare. Its comprehensive search strategy ensured global and diverse methodological representation, enhancing the scope and applicability of the findings. The collaborative review process, involving multiple reviewers, also strengthens the credibility of the analysis.

However, the study has limitations. The initial screening conducted by a single reviewer may have introduced selection biases or errors, despite efforts to mitigate these through team discussions. Additionally, the exclusion of grey literature could have overlooked valuable insights not presented in peer-reviewed journals, potentially limiting the depth of contextual understanding. Moreover, while a quality assessment of included studies was not feasible due to their heterogeneity, the absence of this might have allowed lower-quality studies to influence the overall interpretation of the data.^97^

## Conclusion

This scoping review represents the first comprehensive analysis regarding the epidemiology, aetiology and interventions for medication safety challenges within prison healthcare systems, revealing critical patterns in how institutional constraints shape medication-related risks. Our review observed imbalances in the evidence base, with research disproportionately focused on medication adherence and particular health problems while leaving gaps in understanding of other conditions and wider preventable DRPs. The interplay between security requirements and healthcare delivery was seen to create unique barriers to the delivery of safe healthcare with medicines in the prison environment. While emerging evidence suggests promise through multidisciplinary and pharmacist-led interventions, the limited scope and geographical concentration of existing research constrains our understanding of scalable solutions. Future research must move beyond documenting isolated incidents to developing frameworks that can reconcile security requirements with therapeutic needs while ensuring medication management parallels community standards of care.

## Supplementary material

10.1136/bmjopen-2025-103781 online supplemental file 1

## Data Availability

Data are available upon reasonable request. All data relevant to the study are included in the article or uploaded as supplementary information.
