# Supplementary material for: Exploring medication safety in prisons: a scoping review
Source: BMJ Open. 2026 Mar 23;16(3):e103781. doi: 10.1136/bmjopen-2025-103781 (PMC13034395; doi:10.1136/bmjopen-2025-103781)
Supplement: online supplemental file 1 [file bmjopen-16-3-s001.docx]

**SUPPLEMENTARY FILE S1: COMPLETE SEARCH STRATEGIES**

| **DATABASE** | **RESULTS** | **SEARCH STRATEGY** |
| --- | --- | --- |
| **EMBASE (via Ovid)** | **3,649** | 1. (drug safety OR medication safety OR medication error* OR patient safety OR adverse drug reaction* OR adverse drug event* OR prescribing error* OR medication adherence OR drug compliance OR medication compliance OR non adheren* OR administration error* OR dispensing error* OR drug related problem* OR drug therapy problem* OR medication incident* OR drug incident* OR clinical incident* OR incident report* OR drug error* OR transcription error* OR preventable harm OR avoidable harm OR medication discrepanc* OR drug discrepanc* OR patient harm OR drug adherence OR medication omission* OR drug omission*).mp.  2. (prison* OR jail* OR secur* OR detain* OR detention* OR offender* OR inmate* OR cellmate* OR incarcerat* OR criminal* OR custod* OR defendant*).mp.  3. 1 AND 2 |
| **MEDLINE (via PubMed)** | **632** | ("Drug Safety"[Mesh] OR "Medication Errors"[Mesh] OR "Patient Safety"[Mesh] OR "Drug-Related Side Effects and Adverse Reactions"[Mesh] OR "Medication Adherence"[Mesh] OR "drug safety"[tiab] OR "medication safety"[tiab] OR "medication error"[tiab] OR "adverse drug reaction"[tiab] OR "prescribing error"[tiab] OR "medication adherence"[tiab] OR "non adherence"[tiab] OR "drug related problem"[tiab] OR "medication incident"[tiab] OR "preventable harm"[tiab] OR "medication omission"[tiab])  **AND**  ("Prisons"[Mesh] OR "Prisoners"[Mesh] OR "prison"[tiab] OR "jail"[tiab] OR "inmate"[tiab] OR "incarcerated"[tiab] OR "detention"[tiab] OR "offender"[tiab] OR "custody"[tiab]) |
| **PsycINFO (via Ovid)** | **432** | 1. exp Drug Safety/ OR exp Medication Errors/ OR exp Patient Safety/ OR exp Side Effects (Drug)/ OR exp Treatment Compliance/  2. (drug safety OR medication safety OR medication error* OR patient safety OR adverse drug reaction* OR medication adherence OR non adheren*).mp.  3. 1 OR 2  4. exp Correctional Institutions/ OR exp Prisoners/  5. (prison* OR jail* OR inmate* OR incarcerat*).mp.  6. 4 OR 5  7. 3 AND 6 |
| **CINAHL PLUS (via EBSCOhost)** | **1,919** | S1: (MH "Drug Safety") OR (MH "Medication Errors") OR (MH "Patient Safety") OR (MH "Drug Reactions") OR (MH "Medication Compliance")  S2: TI ( drug safety OR medication safety OR medication error* OR adverse drug reaction* ) OR AB ( drug safety OR medication safety OR medication error* OR adverse drug reaction* )  S3: S1 OR S2  S4: (MH "Prisons") OR (MH "Prisoners")  S5: TI ( prison* OR jail* OR inmate* ) OR AB ( prison* OR jail* OR inmate* )  S6: S4 OR S5  S7: S3 AND S6 |
| **COCHRANE LIBRARY** | **43** | #1: [mh "Drug Safety"] OR [mh "Medication Errors"] OR [mh "Patient Safety"] OR [mh "Medication Adherence"]  #2: (drug safety OR medication safety OR medication error* OR adverse drug reaction*):ti,ab,kw  #3: #1 OR #2  #4: [mh "Prisons"] OR [mh "Prisoners"]  #5: (prison* OR jail* OR inmate* OR incarcerat*):ti,ab,kw  #6: #4 OR #5  #7: #3 AND #6 |
| **WEB OF SCIENCE (Core Collection)** | **6,521** | #1: TS=("drug safety" OR "medication safety" OR "medication error*" OR "patient safety" OR "adverse drug reaction*" OR "adverse drug event*" OR "prescribing error*" OR "medication adherence" OR "drug compliance" OR "medication compliance" OR "non adheren*" OR "administration error*" OR "dispensing error*" OR "drug related problem*" OR "preventable harm" OR "medication omission*")  #2: TS=(prison* OR jail* OR secur* OR detain* OR detention* OR offender* OR inmate* OR incarcerat* OR criminal* OR custod*)  #3: #1 AND #2 |
| **TOTAL** | **13,196** | **After duplicates removed (n=1,800): 11,396 records**  **Title/abstract screened: 11,396 \| Full-text assessed: 105 \| Final included: 42** |

**SEARCH STRATEGY NOTES**

1. Truncation (*) was used to capture word variations (e.g., error* captures error, errors)

2. Boolean operators AND and OR were used to combine concepts appropriately

3. Medical Subject Headings (MeSH) terms were used where available in MEDLINE

4. EMTREE terms were utilized in Embase searches

5. Database-specific syntax was adapted for each platform while maintaining search concept consistency

6. No language restrictions were initially applied during searching; English language filter was applied during screening

7. Date limits were consistently applied across all databases (2000-2023)

8. Grey literature databases were not searched due to resource constraints and study design focus on peer-reviewed literature

**SUPPLEMENTARY FILE S2: PRISMA-ScR CHECKLIST**

Preferred Reporting Items for Systematic reviews and Meta-Analyses extension for Scoping Reviews (PRISMA-ScR) Checklist

| **SECTION** | **ITEM** | **PRISMA-ScR CHECKLIST ITEM** | **REPORTED ON PAGE #** |
| --- | --- | --- | --- |
| **TITLE** |  |  |  |
| Title | 1 | Identify the report as a scoping review. | **1** |
| **ABSTRACT** |  |  |  |
| Structured summary | 2 | Provide a structured summary that includes (as applicable): background, objectives, eligibility criteria, sources of evidence, charting methods, results, and conclusions that relate to the review questions and objectives. | **2-3** |
| **INTRODUCTION** |  |  |  |
| Rationale | 3 | Describe the rationale for the review in the context of what is already known. Explain why the review questions/objectives lend themselves to a scoping review approach. | **4-6** |
| Objectives | 4 | Provide an explicit statement of the questions and objectives being addressed with reference to their key elements (e.g., population or participants, concepts, and context) or other relevant key elements used to conceptualize the review questions and/or objectives. | **6-7** |
| **METHODS** |  |  |  |
| Protocol and registration | 5 | Indicate whether a review protocol exists; state if and where it can be accessed (e.g., a Web address); and if available, provide registration information, including the registration number. | **6-7** |
| Eligibility criteria | 6 | Specify characteristics of the sources of evidence used as eligibility criteria (e.g., years considered, language, and publication status), and provide a rationale. | **10-12** |
| Information sources | 7 | Describe all information sources in the search (e.g., databases with dates of coverage and contact with authors to identify additional sources), as well as the date the most recent search was executed. | **7** |
| Search | 8 | Present the full electronic search strategy for at least 1 database, including any limits used, such that it could be repeated. | **7-8, S1** |
| Selection of sources of evidence | 9 | State the process for selecting sources of evidence (i.e., screening and eligibility) included in the scoping review. | **7-8** |
| Data charting process | 10 | Describe the methods of charting data from the included sources of evidence (e.g., calibrated forms or forms that have been tested by the team before their use, and whether data charting was done independently or in duplicate) and any processes for obtaining and confirming data from investigators. | **12-13, S3** |
| Data items | 11 | List and define all variables for which data were sought and any assumptions and simplifications made. | **13-17** |
| Critical appraisal of individual sources of evidence | 12 | If done, provide a rationale for conducting a critical appraisal of included sources of evidence; describe the methods used and how this information was used in any data synthesis (if appropriate). | **N/A** |
| Synthesis of results | 13 | Describe the methods of handling and summarizing the data that were charted. | **13-15** |
| **RESULTS** |  |  |  |
| Selection of sources of evidence | 14 | Give numbers of sources of evidence screened, assessed for eligibility, and included in the review, with reasons for exclusions at each stage, ideally using a flow diagram. | **14-17** |
| Characteristics of sources of evidence | 15 | For each source of evidence, present characteristics for which data were charted and provide the citations. | **16-17, S1** |
| Critical appraisal within sources of evidence | 16 | If done, present data on critical appraisal of included sources of evidence (see item 12). | **N/A** |
| Results of individual sources of evidence | 17 | For each included source of evidence, present the relevant data that were charted that relate to the review questions and objectives. | **18-32 (Tables 5-7)** |
| Synthesis of results | 18 | Summarize and/or present the charting results as they relate to the review questions and objectives. | **33-41** |
| **DISCUSSION** |  |  |  |
| Summary of evidence | 19 | Summarize the main results (including an overview of concepts, themes, and types of evidence available), link to the review questions and objectives, and consider the relevance to key groups. | **33-41** |
| Limitations | 20 | Discuss the limitations of the scoping review process. | **40-41** |
| Conclusions | 21 | Provide a general interpretation of the results with respect to the review questions and objectives, as well as potential implications and/or next steps. | **41** |
| **FUNDING** |  |  |  |
| Funding | 22 | Describe sources of funding for the included sources of evidence, as well as sources of funding for the scoping review. Describe the role of the funders of the scoping review. | **1** |

JBI = Joanna Briggs Institute; PRISMA-ScR = Preferred Reporting Items for Systematic reviews and Meta-Analyses extension for Scoping Reviews

**SUPPLEMENTARY FILE S3: Standardised data extraction form**

| Title, Authors,  Year | Country | Setting | Study period | Study design | Study population | Aim of study | Outcomes measured | Definitions of terms used e.g.  adherence | Validation  method |
| --- | --- | --- | --- | --- | --- | --- | --- | --- | --- |
|  |  |  |  |  |  |  |  |  |  |

| Data collection  methods | Personnel  collecting data | Epidemiology | Aetiology | Preventable harm  caused | Interventions | Limitations of the study |
| --- | --- | --- | --- | --- | --- | --- |
|  |  |  |  |  |  |  |

**Supplementary Table S1:** **Summary table of included studies**

| **AUTHOR AND YEAR** | **Country of origin** | **Study design** | **Study Aim** | **Medication class(es) studied** | **DRPs studied** | **EPIDEMIOLOGY** | **AETIOLOGY** | **INTERVENTION** |
| --- | --- | --- | --- | --- | --- | --- | --- | --- |
| (35) White BL et al. (2006) | USA | Mixed methods - Interviews | Assessing barriers and facilitators of adherence to ART DOT in prison | Antiretroviral therapy  HIV | Non- adherence | √ |  |  |
| (36) Wohl DA et al. (2003) | USA | Quantitative  Health records | To evaluated infected jail detainees prescribed or eligible for antiretroviral therapy (ART) | Antiretroviral therapy  HIV | Non- adherence | √ |  |  |
| (37) Lobato MN et al. (2005) | USA | Quantitative  Health records | To determine the acceptability, tolerability, and completion of treatment. | TB treatment | ADEs | √ |  |  |
| (38) Baillargeon J et al. (2000) | USA | Quantitative  Health records | To examine compliance with antidepressant medication | Antidepressants | Non- adherence | √ |  |  |
| (39) Saberi P et al. (2012) | USA | Quantitative  Survey | To compare preferences for DOT versus self-administered ART | Antiretroviral therapy  HIV | Non- adherence |  | √ |  |
| (40) White BL et al. (2015) | USA | Quantitative  Health records | To compare DOT vs self-administered ART on adherence and virological outcomes | Antiretroviral therapy  HIV | Non- adherence | √ |  |  |
| (41) Merker A et al. (2018) | USA | Quantitative  Health records | To evaluate the effectiveness of STR and MTR as related to maintaining virologic suppression, adherence, and discontinuation rates of STRs compared to MTRs. | Antiretroviral therapy  HIV | Non- adherence | √ |  |  |
| (42) Roberson DW et al. (2009) | USA | Qualitative  Interviews | To Identify barriers to and facilitators of adherence to ART while in  Prison. | Antiretroviral therapy  HIV | Non- adherence |  | √ |  |
| (43) Kanu LN et al. (2020) | USA | Quantitative  Health records | To evaluate the glaucoma profile of prison inmates  and follow-up metrics. | Glaucoma medications | Non- adherence | √ |  |  |
| (44) Carmenates J et al. (2001) | USA | Quantitative  Health records | To Evaluate the implementation and impact  of an automated check-and-sortation system  in a correctional health care system. | Multiple medication classes | MEs | −−−−−−−−−−−−−−−−−− |  | √ |
| (45) Avery AK et al. (2013) | USA | Quantitative  Health records | To assess engagement in HIV care and ART adherence in jail settings | Antiretroviral therapy  HIV | Non- adherence | √ |  |  |
| (46) Berg CJ et al. (2013) | USA | Quantitative - Survey | To evaluate predictors of adherence to smoking cessation interventions | Nicotine replacement therapy | Non- adherence |  |  | √ |
| (47) Cunningham WE et al. (2019) | USA | Quantitative  Health records | To examine self-reported ART adherence and viral load in criminal justice populations | Antiretroviral therapy  HIV | Non- adherence | √ |  |  |
| (48) Magola-Makina E et al. (2022) | UK | Qualitative - Interviews | To explore challenges to safer prescribing and medication monitoring | Multiple medication classes | PIP/PIM |  | √ |  |
| (49) Abuzour AS et al. (2021) | UK | Mixed methods - Health records & interviews | To implement prescribing safety indicators in prisons | Multiple medication classes | PIP/PIM | √ | √ |  |
| (50) Soni A et al. (2020) | UK | Quantitative  Health records | To study reasons for prescribing and misuse of gabapentinoids | Gabapentinoids | DDI /PIP | √ |  |  |
| (51) Bebbington E et al. (2020) | UK | Quantitative  Health records | To evaluate framework for safe prescribing of psychoactive medications | Psychotropic medication. | PIP/PIM | √ | −−−−−−−−−−−−−−−− |  |
| (52) McFadzean IJ et al. (2023) | UK | Mixed methods - Incident reports | To analyse patient safety incidents in prisons | Multiple medication classes | ADEs | √ | √ |  |
| (53) Duke K et al. (2022) | UK | Qualitative    Interview | To explore problematisations of prescribed medication in prisons | Multiple medication classes | PIP/PIM |  | √ |  |
| (54) Hassan L et al. (2016) | UK | Quantitative - Health records | To assess appropriateness of psychotropic medication prescribing | Psychotropic medications | PIP/PIM | √ |  |  |
| (55) Mills A et al. (2011) | UK | Mixed methods - Health records & interviews | To explore prisoners' experiences of antipsychotic medication | Antipsychotic medications | Non- adherence |  | √ |  |
| (56) Hassan L et al. (2012) | UK | Mixed methods - Interviews & questionnaires | To examine views on prisoners holding their own medications | Multiple medication classes | Non- adherence |  | √ |  |
| (57) Bressington D et al. (2008) | UK | Qualitative  Survey | To assess satisfaction with and adherence to antipsychotic medication | Antipsychotic medications | Non- adherence |  | √ |  |
| (58) Hassan L et al. (2013) | UK | Qualitative  Interviews | To explore perspectives on reasons for psychotropic medication use in prisons | Psychotropic medications | PIP/PIM |  | √ |  |
| (59) Rezansoff SN et al. (2017) | Canada | Quantitative  Health records | To examine adherence to antipsychotic medication and recidivism | Antipsychotic medications | Non- adherence | √ |  |  |
| (60) Dawson KG et al. (2023) | Canada | Quantitative  Health records | To evaluate pharmacist-led interventions for NSAID deprescribing | NSAID medications | PIP/PIM |  |  | √ |
| (61) Milloy MJ et al. (2011) | Canada | Quantitative - Health records | To examine effect of incarceration on HIV treatment adherence | Antiretroviral therapy  HIV | Non- adherence | √ |  |  |
| (62) Small W et al. (2009) | Canada | Qualitative - Interviews | To explore impact of incarceration on HIV treatment adherence | Antiretroviral therapy  HIV | Non- adherence |  | √ |  |
| (63) Farley JD et al. (2005) | Canada | Quantitative    Health records | To assess treatment of chronic hepatitis C in prison | Antiretroviral therapy  HCV | Non- adherence | √ |  |  |
| (64) Soto Blanco JM et al. (2005) | Spain | Quantitative - Questionnaires | To evaluate adherence to antiretroviral treatment | Antiretroviral therapy  HIV | Non- adherence | √ | √ |  |
| (65) Muela A et al. (2020) | Spain | Quantitative – Health records | To promote adherence to psychopharmacological treatment and to analyse whether this increase was similar to that achieved  with TAU. | Psychotropic medications | Non- adherence |  |  | √ |
| (66) Cabelguenne D et al. (2018) | France | Quantitative - Health records | To evaluate benzodiazepine dose reduction program | Benzodiazepine medications | ADRs/PIP |  |  | √ |
| (67) Seyed Alinaghi A et al. (2016) | Iran | Qualitative  Focus group discussions (FGDs) | To assess adherence to ART and TB treatment | Antiretroviral therapy- **HIV**  and **TB** Treatment | Non- adherence |  | √ |  |
| (68) Farhoudi B et al. (2018) | Iran | Qualitative  Focus group discussion (FGDs) | To identify barriers to ART adherence | Antiretroviral therapy  HIV | Non- adherence |  | √ |  |
| (69) Culbert GJ et al. (2019) | Indonesia | Quantitative - Health records | To examine ART adherence associations with methadone | Antiretroviral therapy  HIV | Non- adherence | √ |  |  |
| (70) Culbert GJ et al. (2016) | Indonesia | Mixed methods - Interviews & questionnaires | To examine influence of medication attitudes on ART use | Antiretroviral therapy  HIV | Non- adherence | −−−−−−−−−−−−−−−−−− | √ |  |
| (71) Havnes IA et al. (2014) | Norway | Qualitative - Interviews | To explore control in opioid maintenance treatment | Opioid medications | Non- adherence |  | √ |  |
| (72) Fuge TG et al. (2021) | Ethiopia | Qualitative Interviewing | To explore factors influencing ART initiation | Antiretroviral therapy  HIV | PIP/PIM |  | √ |  |
| (73) Fuge TG et al. (2022) | Ethiopia | Quantitative - Questionnaires | To assess factors affecting ART adherence | Antiretroviral therapy  HIV | Non- adherence | √ | √ |  |
| (74) Ravanholi GM et al. (2019) | Brazil | Quantitative - Health records | To evaluate regular use of ART in prison | Antiretroviral therapy  HIV | Non- adherence | √ |  |  |
| (75) Chan PC et al. (2012) | Taiwan | Quantitative - Health records | To assess TB treatment and adverse events | TB therapy | ADEs | √ |  |  |
| (76) Shalihu N et al. (2014) | Namibian | Qualitative -  Interviewed | to investigate the barriers to  ART adherence in an urban Namibian all-male, maximum  security prison | Antiretroviral therapy  HIV | Non- adherence |  | √ |  |
