# Supplementary material for: Exploring medication safety in prisons: a scoping review
Source: BMJ Open. 2026 Mar 23;16(3):e103781. doi: 10.1136/bmjopen-2025-103781 (PMC13034395; doi:10.1136/bmjopen-2025-103781)
Supplement: Supplementary file 2 [file bmjopen-2025-103781.reviewer_comments.pdf]

PEER REVIEW HISTORY

BMJ Open publishes all reviews undertaken for accepted manuscripts. Reviewers are asked to complete a checklist review form and are provided with free text boxes to elaborate on their assessment. These free text comments are reproduced below.

ARTICLE DETAILS

Title (Provisional)

Exploring medication safety in prisons: A scoping review

Authors

Alsuwat , Mohammed; Shaw, Jennifer; Keers, Richard Neil

VERSION 1 - REVIEW

**Reviewer**	**1**
**Name**	**Nicholls, Gary**
**Affiliation**	**UNSW Sydney, Medicine**
**Date**	**02-Jun-2025**
**COI**	**None**

This is very interesting work, thanks for the opportunity to review it.

There is a lot of work happening (that perhaps isn't formally written up) in medication safety and 'Quality Use of Medicines' (QUM) where I work in Australia. It's a growing area and people who work in prisons worldwide are discussing and working towards this at various international conferences.

Your work will contribute to QUM appreciation worldwide for this vulnerable population and is great to see.

Other future points of discussion may include - access to newer drugs that may complicate polypharmacy and create further interactions / adverse events e.g. ADHD medications, GLP-1 meds etc.

**Reviewer**	**2**
**Name**	**Tsuchiya, Masami**
**Affiliation**	**Keio University Faculty of Pharmacy Graduate School of Pharmacy**
**Date**	**07-Dec-2025**

COI **None**

The authors present a scoping review of medication safety issues in prison settings, addressing an important and under-researched area. The topic is timely, and the manuscript compiles a substantial body of evidence across diverse medication-related domains. The review has several strengths, including broad database coverage and a structured synthesis. However, there are methodological and reporting issues that require clarification or revision before the manuscript can be considered for publication. Major concerns relate to the articulation of the review questions, transparency of the search strategy, clarification of the review scope, and interpretation of adherence measures within the prison context. Additional minor revisions would improve clarity and readability. Detailed comments are provided below.

Major comments

1. The Background section is overly long and would benefit from concise restructuring.

The Introduction provides extensive contextual information about prison healthcare, but several paragraphs do not directly contribute to defining the study's rationale or scope. A more concise and focused Background would improve readability and better support the stated aims of the review.

2. Methods: Research questions are not explicitly articulated.

While the overall study aim is described, the manuscript does not specify the research questions guiding the scoping review. According to PRISMA-ScR and JBI guidance, scoping reviews should clearly state the review question(s) and define the PCC framework.

Explicitly articulating the review questions would strengthen methodological clarity and ensure alignment between the aim and the evidence mapping.

3. Methods: Full search queries should be provided in the supplementary materials.

The search strategy is described only conceptually (databases, time frame, and keyword groups), and Table 1 lists high-level search terms; however, full reproducible search strings for each database are not included.

PRISMA-ScR and BMJ Open require full database-specific search strategies—including field tags, Boolean operators, truncation, and subject headings—to be reported.

Please include the complete search strategies for all databases in a supplemental appendix to ensure reproducibility.

4. Methods: The scope of the review requires further clarification regarding the definition of "medication class" versus "single medication".

The exclusion criteria state that studies focusing on a single medication were excluded.However, several included studies examine relatively narrow therapeutic groups (e.g., antidepressants, antipsychotics, ART, opioid agonist therapy).

While these may fit the authors' intended definition of "broad medication classes," the rationale for classifying them as such is not sufficiently explained. To enhance transparency, the authors should more precisely define how medication classes were distinguished from single medications and describe how these criteria were applied during study selection.

5. Discussion: Interpretation of adherence measures such as MPR does not sufficiently consider prison medication management systems.

Several studies included in the review use MPR or similar measures to assess medication adherence. However, in prison settings, medication distribution systems—such as directly observed therapy (DOT), in-possession policies, or restricted medication handling—can prevent individuals from physically holding their medications even when they are fully adherent. Low MPR values may therefore reflect institutional policies rather than true non-adherence. The Discussion should explicitly address this contextual limitation, as it has substantial implications for interpreting medication safety issues in prisons.

Minor Comment

1. Page 39, Line 667 — Typographical error.

The phrase "this problem of this problem" contains an unintended repetition.

Please revise to "this problem" or "the problem."

VERSION 1 - AUTHOR RESPONSE

REVIEWER 1 COMMENTS

Dr. Gary Nicholls, UNSW Sydney

REVIEWER COMMENT	AUTHOR RESPONSE
This is very interesting work, thanks for the opportunity to review it. There is a lot of work happening (that perhaps isn't formally written up) in medication safety and 'Quality Use of Medicines' (QUM) where I work in Australia. It's a growing area and people who work in prisons worldwide are discussing and working towards this at various international conferences. Your work will contribute to QUM appreciation worldwide for this vulnerable population and is great to see. Other future points of discussion may include - access to	We thank Reviewer 1 for these encouraging comments and valuable insights about emerging therapeutic areas. We agree that access to newer medications like ADHD treatments and GLP-1 agonists presents evolving challenges in prison healthcare, particularly regarding polypharmacy interactions and adverse events. These important considerations will be incorporated into future research directions and publications building on this foundational scoping review.

newer drugs that may complicate polypharmacy and create further interactions / adverse events e.g. ADHD medications, GLP-1 meds etc.	

REVIEWER 2 MAJOR COMMENTS

Dr. Masami Tsuchiya, Keio University Faculty of Pharmacy Graduate School of Pharmacy

REVIEWER COMMENT	AUTHOR RESPONSE
**(Original- Page 4, Lines 95-142)**<br>**Major Comment 1:**<br>The Background section is overly long and would benefit from concise restructuring. The Introduction provides extensive contextual information about prison healthcare, but several paragraphs do not directly contribute to defining the study's rationale or scope. A more concise and focused Background would improve readability and better support the stated aims of the review.	Many thanks for this comment. We have substantially condensed the Background section while retaining essential contextual information. We removed redundant text from paragraphs and streamlined the flow to focus specifically on: (1) the unique challenges of prison healthcare, (2) the significance of medication safety issues, and (3) the knowledge gap our study addresses. The revised Background is approximately **30% shorter** and maintains better focus on the study rationale
**(Original- Page 7, after line 168)**<br>**Major Comment 2:**<br>Methods: Research questions are not explicitly articulated. While the overall study aim is described, the manuscript does not specify the research questions guiding the scoping review. According to PRISMA-ScR and JBI guidance, scoping reviews should clearly state the review question(s) and define the PCC framework.	We have **added** explicit research questions using the PCC framework as recommended (page 6, Lines 129-140 in the **revised manuscript**):<br><br>- Population: Prisoners and incarcerated individuals<br>- Concept: Drug-related problems including their nature, frequency, contributory factors, and interventions<br>- Context: Prison environments<br><br>Our specific research questions are:<br>1. What is the nature and frequency of drug-related problems in prison healthcare settings?<br>2. What are the contributory factors associated with drug-related problems in prison healthcare settings? 3. What interventions have been implemented and evaluated to improve medication safety in prison healthcare?

**(Supplementary File 1):**<br>**Major Comment 3:**<br>Methods: Full search queries should be provided in the supplementary materials. The search strategy is described only conceptually (databases, time frame, and keyword groups), and Table 1 lists high-level search terms; however, full reproducible search strings for each database are not included. PRISMA-ScR and BMJ Open require full database-specific search strategies.	We have created a comprehensive supplementary file (**Supplementary File 1**: "Complete Search Strategies") containing the full database-specific search strategies for all six databases. This includes: complete search strings with field tags ([ti], [ab], [kw]), Boolean operators (AND, OR) and truncation (*), subject headings (MeSH terms, EMTREE terms), all filters and limits applied, and number of results from each database. This supplementary file ensures full reproducibility of our search methodology.
**(Original- Inserted after Table 3, line 200)**<br>**Major Comment 4:**<br>Methods: The scope of the review requires further clarification regarding the definition of "medication class" versus "single medication". The exclusion criteria state that studies focusing on a single medication were excluded. However, several included studies examine relatively narrow therapeutic groups (e.g., antidepressants, antipsychotics, ART, opioid agonist therapy).	We have clarified our inclusion criteria by **adding** the following definition in the Methods section (page 11, Lines 170-178 in the **revised manuscript**):<br><br>"For this review, 'broad medication classes' were defined as therapeutic categories encompassing multiple individual medications with similar mechanisms of action or clinical applications (e.g., antidepressants including SSRIs, tricyclics, etc.; antipsychotics including typical and atypical agents). Antiretroviral therapy was considered a broad medication class as HIV treatment typically involves combination therapy with multiple drug classes. Similarly, opioid agonist therapy encompasses multiple agents (e.g., methadone, buprenorphine, naltrexone) used in opioid substitution treatment. Studies examining single medications only (e.g., amoxicillin, metformin) were excluded as they would not provide generalisable insights about medication class-level safety challenges in prison settings."
**(Original- Page 38, after line 635)**<br>**Major Comment 5:**<br>Discussion: Interpretation of adherence measures such as MPR does not sufficiently consider prison medication management systems. Several studies included in the review use MPR or similar measures to assess medication adherence. However, in prison settings, medication distribution systems—such as directly observed therapy (DOT), in-possession policies, or restricted medication handling—can prevent individuals from physically holding their medications even when they are fully adherent. Low MPR values may	This is an excellent point that we had not adequately addressed. We have **added** the following in the Discussion section (page 37, line 614 in the **revised manuscript**):<br><br>" An important methodological consideration when interpreting adherence data from prison settings relates to how institutional medication management systems may affect adherence measurement validity. Multiple studies in our review assessed medication adherence using various community-based measures including MPR[59], self-reported adherence questionnaires and scales[36,40,47], pill counts[36], electronic monitoring caps[36], and pharmacy refill records[40,69,73]. However, some adherence

therefore reflect institutional policies rather than true non-adherence.	measures may be associated with limitations when applied in prison environments where directly observed therapy (DOT)[35,36,39,40], in-possession policies[56], or restricted medication handling can be common practice. Reported adherence rates may therefore be reflective of institutional policies than actual patient adherence. For example, in correctional facilities, as in hospitals, patients do not refill prescriptions and with exception of medicines kept 'in possession', healthcare staff control medication administration according to security protocols. Some medications may also be kept as 'stock' in clinic rooms rather than as named dispensing to individual patients, which may make tracking adherence via individual patient dispensing records more challenging.[35,36] Known issues with medication trading and abuse in prison settings are another complicating factor, with concerns about diversion potentially affecting both prescribing decisions and adherence measurement approaches[39,53].<br>Research in correctional settings has demonstrated that even when DOT is implemented, different adherence measurement methods yield different results for the same patients, with medication administration records showing higher adherence than electronic monitoring caps for identical medication regimens.[36,40] Future research in correctional settings should consider the impact of institutional medication policies when developing and selecting adherence measures, potentially favouring methods that account for these and which reflect actual medication usage patterns by patients."

REVIEWER 2 MINOR COMMENT

REVIEWER COMMENT	AUTHOR RESPONSE
**Minor Comment:**<br>**(Original- Page 39**, **Line 666** — Typographical error. The phrase "this problem of this problem" contains an unintended repetition. Please revise to "this problem" or "the problem."	Thank you for catching this error. We have corrected "this problem of this problem" to "this problem".

SUMMARY OF CHANGES

The following key changes have been made to strengthen the manuscript:

1. Revised "Strengths and limitations" section to five concise, methods-focused bullet points
2. Removed "What This Paper Adds" section from Introduction
3. Condensed Background section for improved focus and readability
4. Added explicit research questions using PCC framework in Methods section
5. Clarified definition of "medication class" vs "single medication" in inclusion criteria
6. Added comprehensive discussion of MPR measurement limitations in prison settings
7. Created detailed supplementary files for complete search strategies and PRISMA-ScR checklist
8. Fixed typographical error on line 666

We believe these revisions have significantly improved the methodological clarity, reproducibility, and interpretive accuracy of our scoping review.

VERSION 2 - REVIEW

**Reviewer**	**2**
**Name**	**Tsuchiya, Masami**
**Affiliation**	**Keio University Faculty of Pharmacy Graduate School of Pharmacy**
**Date**	**12-Feb-2026**
**COI**	

Thank you for the opportunity to review this insightful article. The authors have addressed my comments satisfactorily, and I have no major concerns remaining.

VERSION 2 - AUTHOR RESPONSE

Reviewer 2 (Dr. Masami Tsuchiya, Keio University)

> *Thank you for the opportunity to review this insightful article. The authors have addressed my comments satisfactorily, and I have no major concerns remaining.*

Response: We thank Reviewer 2 for their positive assessment and for confirming that all previous comments have been satisfactorily addressed. No further changes were required in response to this reviewer.
